# Histomorphometric Analysis of Differential Regional Bone Regeneration Induced by Distinct Doped Membranes

**DOI:** 10.3390/polym14102078

**Published:** 2022-05-19

**Authors:** Manuel Toledano, Cristina Vallecillo, Aida Gutierrez-Corrales, Daniel Torres-Lagares, Manuel Toledano-Osorio, María-Angeles Serrera-Figallo

**Affiliations:** 1 Faculty of Dentistry, Colegio Máximo de Cartuja s/n, University of Granada, 18071 Granada, Spain; toledano@ugr.es (M.T.); cvallecillorivas@hotmail.com (C.V.); 2 Faculty of Dentistry, Oral Surgery Section, University of Sevilla, Avicena s/n, 41009 Sevilla, Spain; agcorrales@us.es (A.G.-C.); danieltl@us.es (D.T.-L.); maserrera@us.es (M.-A.S.-F.)

**Keywords:** zinc, doxycycline, silica, guided bone regeneration, bone, osteoid, polymer, membrane

## Abstract

Our objective is to evaluate the regional regenerative potential of calvarial bone in critical-sized defects in a rabbit model using novel nanostructured silica-loaded membranes doped with zinc or doxycycline. Nanostructured membranes of (MMA)_1_-co-(HEMA)_1_/(MA)_3_-co-(HEA)_2_ loaded with 5 wt% of SiO_2_ nanoparticles (HOOC-Si-Membranes) were doped with zinc (Zn-HOOC-Si-Membrane) or doxycycline (Dox-HOOC-Si-Membrane). Critical bone defects were created on six New-Zealand-breed rabbit skulls and covered with the membranes. A sham defect without a membrane was used as the control. After six weeks, a histological analysis (toluidine blue technique) was employed to determine the area percentages of newly formed bone, osteoid bone, and soft tissue. The measurements were performed by dividing the total defect area into top (close to the membrane) and bottom (close to the dura mater) regions, or peripheral (adjacent to the old bone) and central (the sum of the remaining zones) regions. The peripheral regions of the defects showed higher osteogenic capacity than the central areas when the membranes were present. The proportion of new bone adjacent to the dura was similar to that adjacent to the membrane only when the HOOC-Si-Membranes and Zn-HOOC-Si-Membranes were used, indicating a direct osteoinductive effect of the membranes.

## 1. Introduction

Guided bone regeneration (GBR) is a consolidated treatment modality to accomplish bone regeneration, particularly in the maxillofacial region to gain bone volume [1] facing the newly forming bone in the underlying defect [2]. The disposal of enough bone dimension plays a pivotal role not only in maintaining aesthetics but also for functional purposes. The available amount of alveolar bone conditions the long-term behavior of dental implants [3].

GBR treatment is based on the application of bone substitute materials frequently used in conjunction with barrier membranes to cover an osseous defect [4]. Traditionally, membranes have been considered to act as a passive element excluding selective tissues, but the potential role of the membrane and the membrane material properties as bioactive modulators for regeneration procedures have been underestimated [2]. A membrane may, per se, create an optimal environment that promotes bone formation even in the absence of scaffolding materials [5,6]. The GBR concept is based on the hypothesis that the membrane performs an occlusive function excluding, at the local host location, nonosteogenic tissues from interfering with bone healing [7,8]. Hence, the bone wound-space can only be repopulated with osteogenic cells coming from the adjacent peripheral bone tissue [8]. Although the GBR concept is generally assumed, the underlying biological mechanisms are as yet insufficiently explained, especially in the central and peripheral regions of defects.

Membranes based on nonresorbable materials such as expanded polytetrafluoroethylene (e-PTFE) constitute the first generation of barrier membranes. These membranes are characterized by maintaining structural integrity during healing, as well as good biocompatibility. Nonresorbable membranes have mostly shown positive results, both in research with experimental studies and in some clinical application procedures [9,10]. However, these membranes have the great disadvantage of requiring additional surgery to remove them once the healing time has elapsed [11]. A second generation of resorbable, synthetic or natural, membranes was, therefore, developed, aiming to avoid the need for the extra surgical procedure [12]. Several biodegradable materials have been tested with varying success in GBR, ranging from collagen type I to different copolymers of polylactic and polyglycolic acid [8] with unpredictable clinical outcomes.

Novel nonresorbable composite membranes based on electrospun fibers of a mixture of (MMA)_1_-co-(HEMA)_1_ and (MA)_3_-co-(HEA)_2_ were synthesized in order to enhance hydrophilicity, mechanical properties, cell–membrane interactions, and osteogenic properties, as well as to confer antibacterial properties [13]. Previous morphological characterizations of these matrices were undertaken by atomic force and scanning electron microscopy, and they were mechanically probed by a nanoindenter. Biomimetic calcium phosphate precipitation on these polymeric tissues was also assessed. Cell viability tests were performed using oral mucosa fibroblasts and osteoblasts. Zinc loading on the matrices did not modify their morphologies but increased nanomechanical properties and decreased nanoroughness. Biomimetic precipitation of calcium and phosphate on the matrix surfaces was observed in these zinc-loaded specimens. The matrices were found to be nontoxic to cells. Silica doping of the membranes enhanced osteoblast proliferation and bioactivity and increased mechanical properties. The membrane morphologies and mechanical properties were similar to those of trabecular bone. Zinc and doxycycline doping did not exert changes, but they increased novel membrane bioactivity. At short time points after seeding, cells in silica and zinc- or doxycycline-doped membranes were firmly attached to experimental tissues through filopodia. The cells produced collagen and minerals onto the surfaces [14]. The inclusion of silica nanoparticles (SiO_2_-NPs), as well as adding zinc and doxycycline to the membrane’s formulation, improved the bone formation potential of these novel membranes [15]. They also contributed to trigger not only osteogenic but vascular [16] responses and to increase osteoid new bone formation [17]. Future research will focus on the association between membrane-induced remodeling activities and regenerative bone maturation and how this may result in better bone quality for titanium implant placement and integration [2].

Bone regeneration takes place predictably following GBR application in critical-sized calvarial defects. The bone formation process is achieved via a standard basic pattern that closely resembles bone development and growth during intramembranous ossification. A defect can be assumed as critical when it cannot close spontaneously and depends on specific bone and subject [8]. Historically, within the field of GBR, the objective of most studies has focused on the final volume of newly formed bone thanks to the GBR principle [2]. Determining the distinct effect of the membrane on the structural integrity within the bone defect at different distances from the membrane is essential. This is justified by the fact that these activities have been, classically, analyzed in the entire defect site, which may mask possible differences in specific regions of the defect [18]. The spatial events of healing around the membrane and in the membrane itself should also be determined in order to provide structural evidence of bioactivity. Therefore, osteogenic activity and remodeling to bone repair sites should be explored in the local host environment materials [5,6]. Histomorphometric analysis has generally been used to study bone remodeling and bone structure changes [19]. Thereby, the amount of central and peripheral new bone production and, thus, the different osteogenic potentials between the dura and the pericranium should be quantified separately to further characterize the regenerative contribution of each region of the calvarial defect. The concept of a critical-sized defect is much better evaluated by a quantitative histometric assessment of new bone formation rather than by a subjective measure of “healed” vs. “nonhealed” defects. Our objective is to assess the regional regenerative potential of calvarial bone in critical-sized defects in a rabbit model using novel nanostructured silica-loaded membranes doped with zinc or doxycycline. The null hypothesis to be proved is that the silica-based novel membranes doped with zinc and doxycycline do not facilitate similar bone regeneration in the distinct regions of the defect.

## 2. Materials and Methods

### 2.1. Preparation of Membranes

Nanostructured membranes were manufactured by NanomyP^®^ (Granada, Spain) using an innovative polymeric blend: (MMA)_1_-co-(HEMA)_1_/(MA)_3_-co-(HEA)_2_ 50/50 wt doped with 5 wt% of SiO_2_-NPs. For 2 h, the membranes were incubated in a sodium carbonate buffer solution (333 mM; pH = 12.5) in order to activate them with carboxyl groups (HOOC-Si-Membrane). The carboxyl group disposition on the surface of the artificial tissue and the partial hydrolysis of ester bonds made functionalization possible [20]. Then, the membranes were rinsed with distilled water and dried in a vacuum oven [21]. Immediately after drying, the nanostructured membranes were functionalized with zinc using the ability of the carboxyl groups to complex divalent cations. Doxycycline (Dox) was fixed on the membranes by acid–base interactions between the amino groups of Dox and the membrane carboxyl groups. Aiming to achieve this, the HOOC-Si-Membranes were immersed under continuous stirring at room temperature in aqueous solutions (pH = 7) of both 330 mg L^−1^ of ZnCl_2_ and 800 mg L^−1^ of Dox [22]. Three different membranes were designed: (1) a SiO_2_-NP-doped membrane (HOOC-Si-Membrane), (2) a SiO_2_-NP-doped membrane functionalized with Zn (Zn-HOOC-Si-Membrane), and (3) a SiO_2_-NP-doped membrane functionalized with Dox (Dox-HOOC-Si-Membrane). The membranes were then placed at the bottom of a 24-well plate (Falcon, Becton Dickinson Labware, Franklin Lakes, NJ, USA) and sterilized using an ultraviolet radiation sterilization desk (J.P. SELECTA, Barcelona, Spain).

### 2.2. Animal Experimentation

For this research, six New-Zealand-breed experimental white rabbits with identical characteristics (weighing 3.5–4 kg and aged 6 months) were selected. The rabbits were adequately sheltered; daily *ad libitum* food and water were provided by Rabbit Maintenance Harlan Teklad Lab Animal Diets (2030). The experiment was developed according to the US National Institute of Health (NIH for the Care and Use of Laboratory Animals) and European Directive 86/609/EEC guidelines concerning animals care and use for experimentation. This study also accomplished the European Directive 2010/63/EU concerning animal protection for scientific purposes and all the local laws and regulations. Approval from the Ethics Committee of the institution (CCMI-Ref026/18) was obtained. For ethical reasons, as required by the legislative framework, the minimum number of animals was used. Concerning animal experimentation and histological methods, comparable models have been published [17].

### 2.3. Surgical Procedure

**Figure 1:**
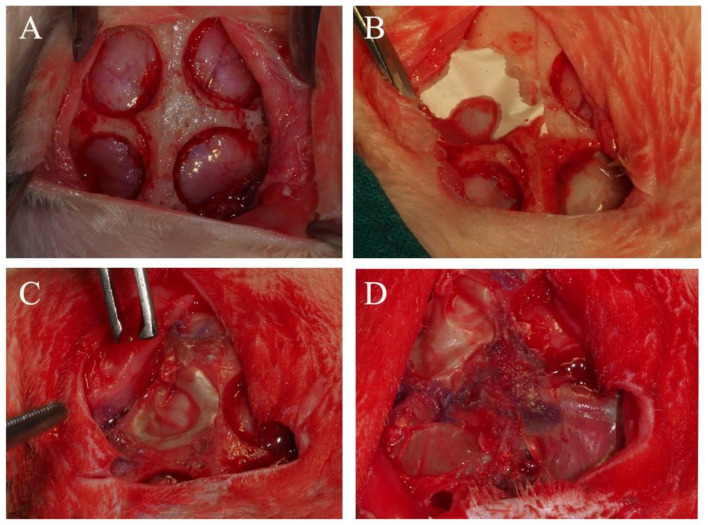
(**A**) Four critical defects were practiced at the parietal bone on each side of the skull midline, permitting the observation of the *dura mater*. (**B**) A membrane was placed on a calvarial defect. (**C**) Two membranes covered two of the four defects. (**D**) Three membranes were placed; the sham group may also be observed.

Before starting the surgery, the animals’ vital signs were obtained, and then they were immobilized. The rabbits were anesthetized using both Propofol (5 mg/kg) and Midazolam (0.25 mg/kg) infiltrated intravenously for induction with 2.8% inspired sevoflurane gas inhalation. Ketorolac (1.5 mg/kg) and tramadol (3 mg/kg) provided the analgesia. Once the animals were sedated and prepared, incisions between their eyes and their ear bases with a number 15 scalpel blade were made. A triangular field was formed when connecting the two incisions with another one in the skull midline. The bone surface was exposed after the connective, muscular, and epithelial tissues were detached from the operation field using a Prichard periosteotome. Then, the skull surface was washed with sterile saline solution. Four critical bone defects were made on the parietal bone: two on each side of the skull midline separated 3 mm from each other. The defect dimensions were 8 mm in diameter and 3 mm in depth, and they were prepared using a trephine (Helmut-Zepf Medical Gmbh, Seitingen, Germany) coupled with an implant micromotor operating at 2000 rpm under saline irrigation. The trephine presented an external diameter of 8 mm, a length of 30 mm, and teeth of 2.35 mm. Piezo surgery was handled to remove the inner table and the medullary bone in every defect. In order to control the depth, a periodontal probe was used. Three bone defects were covered by randomly allocated membranes. The fourth bone defect was not covered (sham group) (Figure 1). To generate the randomization sequence, a specific software (Research Randomizer, V. 4.0, Urbaniak GC and Plous S, 2013, Middletown, CT: Social Psychology Network, USA) was used. Tissucol (Baxter, Hyland S. A. Immuno, Rochester, MI, USA), a fibrin tissue adhesive, was used to immobilize the membranes, and it was applied on the bone rims adjacent to the defects. Once the flaps were repositioned, no mobility and proper adhesion of the studied membranes was confirmed. Sutures were made using resorbable material, making simple stitches as close as possible to the edge in the following sequence: periosteal (4/0), subepidermal (4/0), and skin (2/0). In order to clean the wound, a sterile saline solution was applied. Carprofen (1 mL/12.5 kg) and buprenorphine (0.05 mg/kg) were administered for anti-inflammatory analgesia. Six weeks after surgery, the animals were euthanized with an intravenous overdose of potassium chloride solution. After the procedure, the obtained tissue samples were cut and separated individually [22].

### 2.4. Histology and Histomorphometry

**Figure 2:**
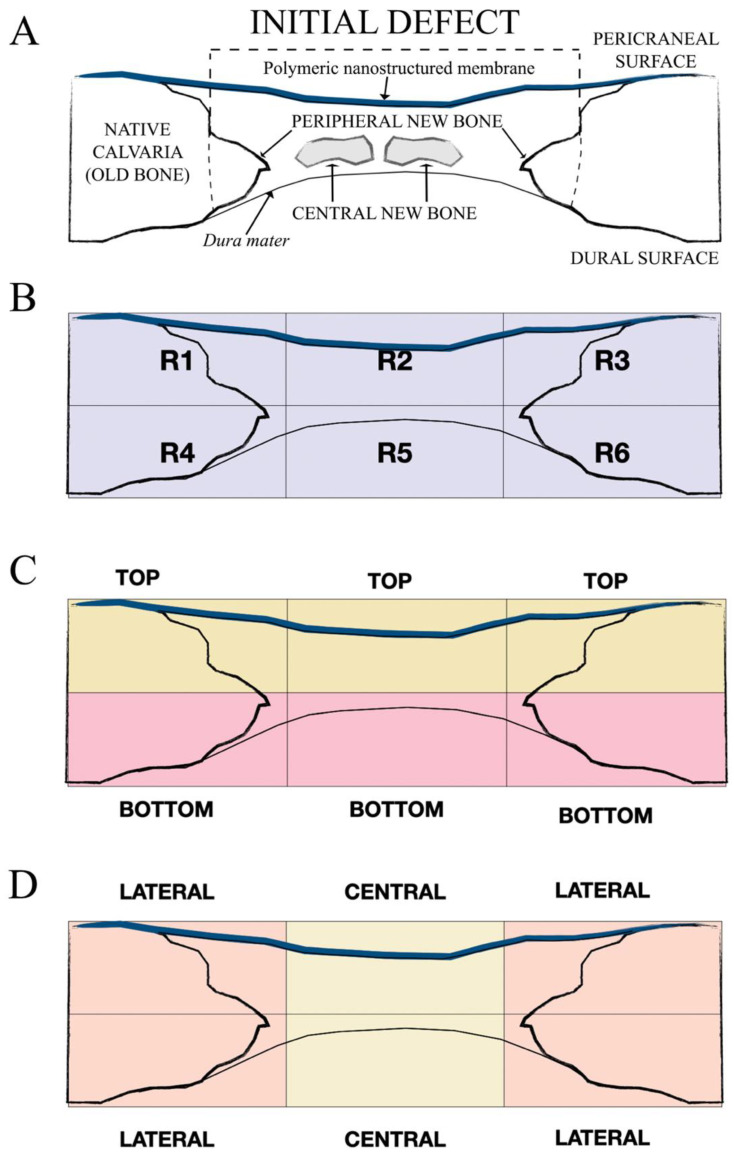
Schematic diagrams showing the defect and the areas of measurement for histomorphometry. (**A**) Histometric analysis of the new bone within the calvarial defect distinguishing between peripheral new bone in continuity with the native calvarial (old bone) and central new bone not in continuity with the calvarial edges. A software rectangular grid consisting of six regions (**B**) covered the entire area of the defect. Top (close to the membrane area; R1, R2, and R3 in B) and bottom (close to the *dura mater*; R4, R5, and R6 in B) areas are represented in (**C**). Lateral or peripheral (adjacent zone to the old bone or native calvarial; R1, R3, R4, and R6 in B) and central (sum of the remaining areas; R2 and R5 in B) zones are represented in (**D**).

The samples were prepared from each rabbit skull by cutting them on the anatomical sagittal plane. To fix the undecalcified bone, a 5% buffered formaldehyde solution (pH 7.4) was employed. The blocks with regenerated bone defects were retrieved using an oscillating autopsy saw (Exakt, Kulzer, Wehrheim, Germany). Subsequently, the dissected specimens were immersed in a 4% formaldehyde and 1% calcium solution, included in acrylic resin, and prepared for ground sectioning. All the sections were coded, and a light microscope (Nikon, Tokyo, Japan) was used to blindly evaluate the histology and histomorphometry. For rapid-contrast tissue analysis, metachromatic dye and histological staining (Merck Toluidine Blue-Merck, Darmstadt, Germany) were employed with a 1% toluidine blue (TB) solution (pH of 3.6) adjusted with HCl. The samples were exposed to the dye with distilled water and air-dried for 10 min at room temperature (23.0 ± 1.0 °C). An Eclipse LV100 microscope (Nikon, Tokyo, Japan) with 20× and 5× lenses was employed to visualize bone from the toluidine morphometric studies. A DSPDS-Fi1 camera (Nikon, Tokyo, Japan) with NIS Elements BR 4.0 software (Nikon, Tokyo, Japan) was used to obtain pictures. In each defect, the area percentages of new bone area (NBA), osteoid area (OA), and soft tissue area (STA) were calculated [13,17]. The measurements were assessed for the total defect area level, as well as by dividing this area into regions that constituted top and bottom regions or central and peripheral regions (Figure 2). A software grid consisting of six zones was employed to divide the total defect area into regions for measurement. The top region, the sum of the top three zones of the grid, was the area close to the membrane. The bottom region, the combination of the bottom three zones of the grid, was the area close to the *dura mater*. Furthermore, the total area of the zones close to the old bone was considered as the peripheral region of the defect, whereas the central region was the combination of the remaining zones [5,6]. The newly formed bone area was determined separately in every zone, and the area percentage of the bone was then calculated with respect to the total defect area or to the area of the respective region. Image analyses were performed using Image J software (National Institutes of Health, Bethesda, MD, USA). For each bone defect, four images were obtained and analyzed.

### 2.5. Statistical Analysis

The means and standard deviations (SDs) were obtained. A nonparametric Friedman test was used for variance analysis, and a nonparametric pairwise comparison of the Friedman rank sums method for post-hoc analysis was employed. The level of significance was set at *p* ≤ 0.05. The assessments were undertaken by means of IBM SPSS Statistics v.24 software (IBM Corporation, Armonk, NY, USA).

## 3. Results

The sites healed uneventfully during retrieval surgery under macroscopic observations. During the experimental period, no visual manifestations or inflammations of surgical complications were determined.

**Figure 3:**
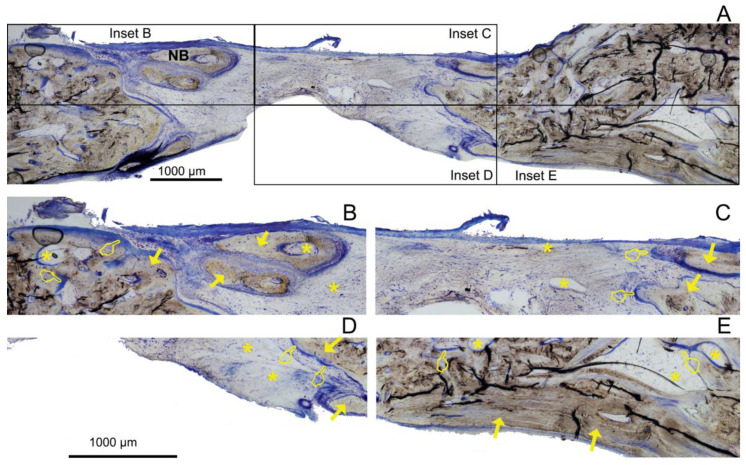
Bone histology obtained after using HOOC-Si-loaded membranes dyed with toluidine blue to visualize mineralized bone at 6 weeks of healing time. Single arrows point to the presence of new bone area (NBA). Osteoid area (OA) is signaled by pointers, and soft tissue area (STA) is indicated by asterisks. (**A**) General view. NB—new bone. Insets AB, AC, AD, and AE reflect the corresponding areas analyzed in Figure 3B–E; (**B**) indicates a partial view of R1 area (top left); (**C**) R2 (top central); (**D**) R5 (bottom central); and (**E**) R6 (bottom right). (**B**) shows an extended area of homogeneous fringe in a clear blue color between the aligned osteoblasts and the mature bone (the osteoid bone at R1). (**C**) permits the observation of the osteoid bone when first deposited and not yet mineralized, signaling the zone where the new bone is growing through at R2. (**D**) reveals the lowest NBA value and the highest STA value that were observed in R5. (**E**) A new bone base at the bottom right of the defect was created next to the *dura mater* in R6.

**Figure 4:**
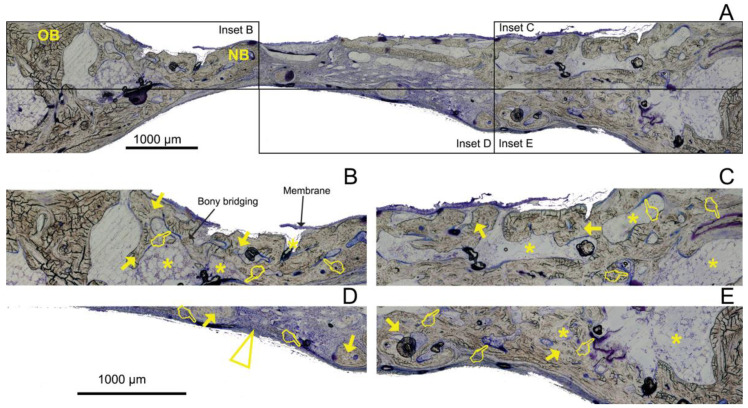
Bone histology obtained after using Zn-HOOC-Si-loaded membranes dyed with toluidine blue to visualize mineralized bone at 6 weeks of healing time. Single arrows point to the presence of new bone area (NBA). Osteoid area (OA) is signaled by pointers, and soft tissue area (STA) is indicated by asterisks. Arrowhead indicates osteons. (**A**) General view. OB—old bone; NB—new bone. Insets AB, AC, AD, and AE reflect the corresponding areas analyzed in Figure 4B–E; (**B**) indicates a partial view of R1 area (top left); (**C**) R3 (top right); (**D**) R5 (bottom central); and (**E**) R6 (bottom right). (**B**) shows the zone with the highest NBA value observed; a bony bridging formed, and membrane is displayed (R1). (**D**) characterizes one of the lowest NBAs analyzed (R5, central adjacent to the dura mater). (**A**,**C**,**E**) permit the observation that osteoid bone primarily occupied the lateral areas of the defect. Soft tissue was present in a lower proportion when compared with the rest of the groups after using this zinc-doped membrane.

**Figure 5:**
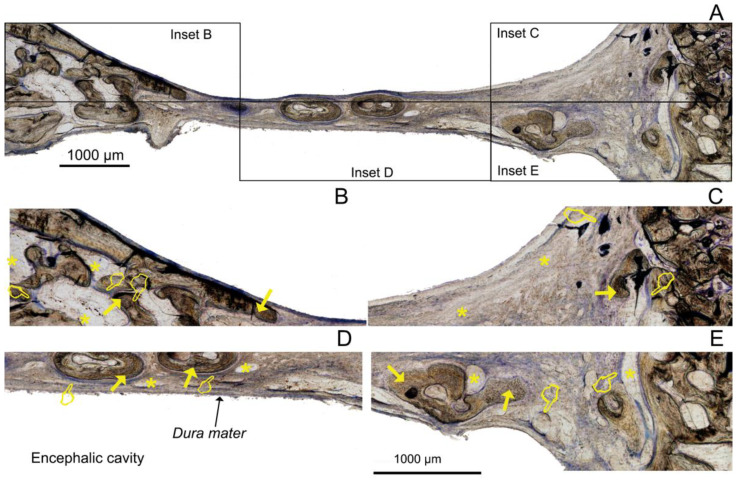
Bone histology obtained after using Dox-HOOC-Si-loaded membranes dyed with toluidine blue to visualize mineralized bone at 6 weeks of healing time. Single arrows point to the presence of new bone area (NBA). Osteoid area (OA) is signaled by pointers, and soft tissue area (STA) is indicated by asterisks. (**A**) General view. Insets AB, AC, AD, and AE reflect the corresponding areas analyzed in Figure 5B–E; (**B**) indicates a partial view of R1 area (top left); (**C**) R3 (top right); (**D**) R5 (bottom central); and (**E**) R6 (bottom right). (**B**,**C**) show zones with the lowest NBAs observed (R1). (**D**) permits the observation of soft tissue that was present in higher proportion when compared with the rest of the groups after using this doxycycline-doped membrane. (**E**) shows a relatively low proportion of STA and a high proportion of NBA in comparison with the rest of the regions within this group of analysis.

**Figure 6:**
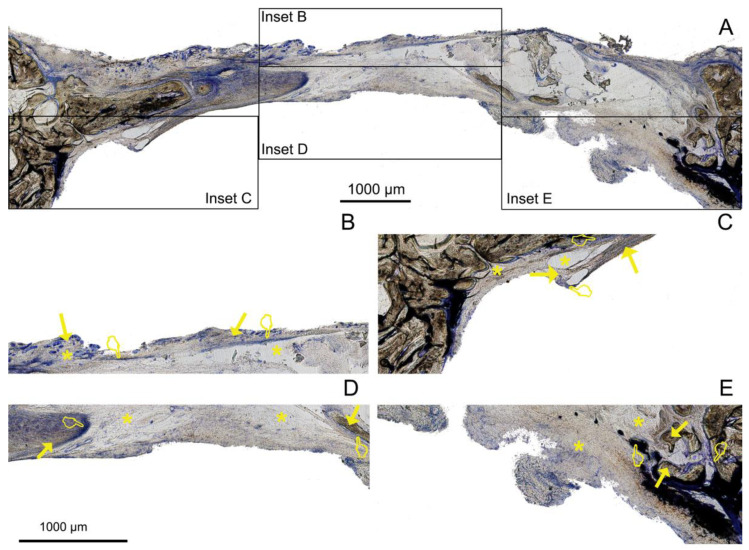
Bone histology obtained analyzing the defects performed in the control (sham) group (no membrane) dyed with toluidine blue to visualize mineralized bone at 6 weeks of healing time. Single arrows point to the presence of new bone area (NBA). Osteoid area (OA) is signaled by pointers, and soft tissue area (STA) is indicated by asterisks. (**A**) General view. Insets AB, AC, AD, and AE reflect the corresponding areas analyzed in Figure 6B–E; (**B**) indicates a partial view of R2 area, (top central); (**C**) R4 (bottom left); (**D**) R5 (bottom central); and (**E**) R6 (bottom right). (**B**) reflects the low and high proportions of new bone and soft tissue, respectively, in the R2 sample. (**C**) shows high new bone created in the peripheral bottom zone of the defect (bottom left). (**D**,**E**) disclose the scarce and abundant new bone created, respectively, at the central and external zones of the defect in the sham group.

The histological examinations showed a substantial amount of bone formed in all the defects, specifically in the membrane groups. There was an increasing presence of trabecular woven bone that emerged from the lateral walls toward the center of the defect (Figure 3, Figure 4 and Figure 5). The bone appeared mature and well-mineralized, as judged by the toluidine blue staining, showing comparable intensity between the old and new bone (Figure 4A). The sham defect presented fewer bone trabeculae of mature bone, bordered endosteally by the bone marrow and periosteally by the overlying soft tissue. The contour of the original bone was almost not restored, soft tissue ingrowth was clearly observed inside the defect, and the newly formed (woven) bone was seldom detected centrally (Figure 6A). On the contrary, the membrane-treated defects revealed a large amount of mature bone, as well as a higher degree of defect restitution (Figure 3, Figure 4 and Figure 5). Structures resembling bone-forming units (osteons) were observed (Figure 4D).

**Figure 7:**
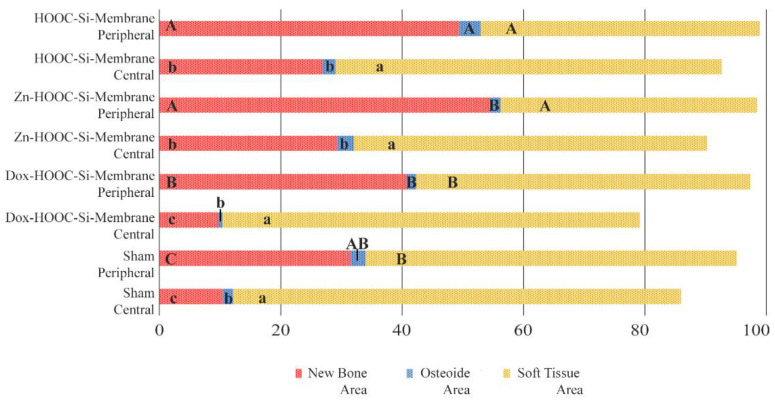
Histomorphometric study of the new bone area (NBA, in red), osteoid area (OA, in blue), and soft tissue area (STA, in yellow) fractions when lateral and central areas of the defect were compared. Same letters within each color indicate no significant differences. Statistical significance is indicated with capital letters for differences among peripheral regions of the distinct membranes and with lower-case letters for differences among central regions of the distinct membranes.

The relative proportion of the woven bone was determined in the total defect area, as well as in different regions of the defects. The new bone was preferentially localized at the periphery rather than in the center of the defects (Figure 4A). The newly formed trabeculae were covered by a periosteum-like tissue (Figure 3A). The bridging of the new bone, when formed, took place under the membrane surface in the outer layer of the defect (Figure 4B). The topological measurements showed a higher proportion of NBA at the periphery (calculated per the peripheral area) compared with the bone proportion found centrally in the respective central areas in all membrane groups except in the sham group, which performed similarly (Figure 6 and Figure 7). The OA values were not different between the periphery vs. the central areas in all the groups, except when the HOOC-Si-Membrane groups were analyzed, where peripheral locations showed higher values than the central areas (Figure 7, Table S1). Both the HOOC-Si-Membranes and the Zn-HOOC-Si-Membranes showed similar STA percentages when the peripheral vs. central regions were compared. Both Dox-Si-Membranes and the sham group attained lower proportions of soft tissue at the central areas than at the peripheral regions of the defect (Figure 7).

**Figure 8:**
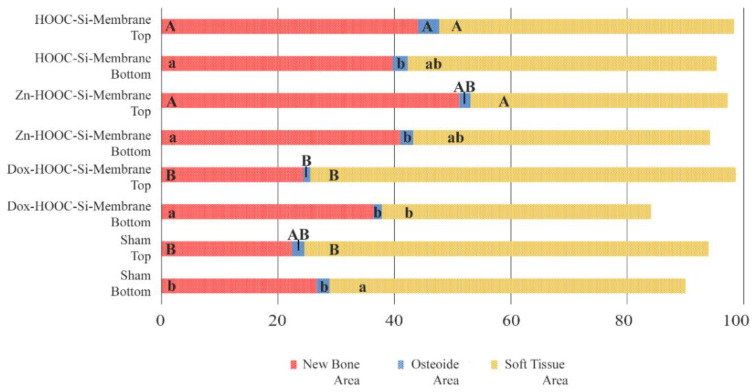
Histomorphometric study of the new bone area (NBA, in red), osteoid area (OA, in blue), and soft tissue area (STA, in yellow) fractions when top and bottom areas of the defect were compared. Same letters within each color indicate no significant differences. Statistical significance is indicated with capital letters for differences among top regions of the distinct membranes and with lower-case letters for differences among bottom regions of the distinct membranes.

In most of the sections, the NBA adjacent to the *dura mater* or at the bottom attained the same proportion as that adjacent to the membrane or at the top region of the defect in all the groups, including the sham group (Figure 8), except in the groups of doxycycline that showed an increase in new bone in the contiguous zone to the *dura mater*. The percentages of OA were similar in all the groups when the top and bottom regions were analyzed, except in the case of defects treated with HOOC-Si-Membranes, where the top regions showed higher osteoid bone values than the bottom areas adjacent to the *dura mater* (Figure 8, Table S2). The OAs were similar in all the groups (Figure 8). The STA was higher at the top regions than at the bottom of the defects in animals that were treated with Dox-HOOC-Si-Membranes, and it was similar at both locations in the rest of the groups (Figure 8).

**Figure 9:**
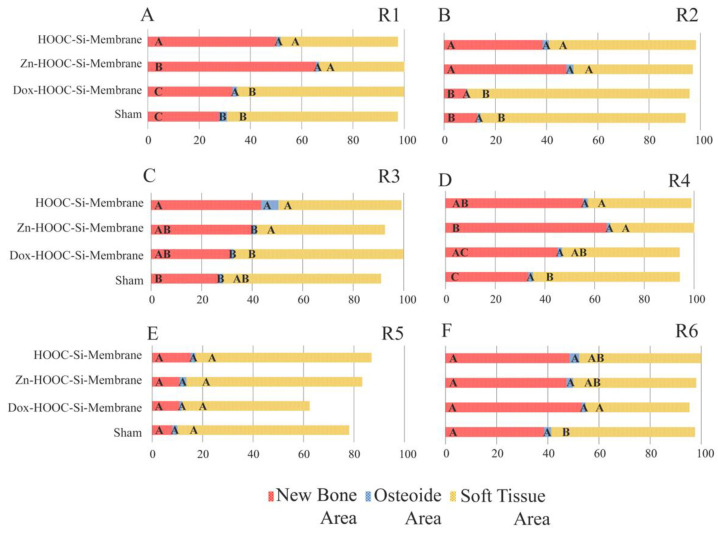
Histomorphometric study of the new bone area (NBA, in red), osteoid area (OA, in blue), and soft tissue area (STA, in yellow) fractions in each of the six regions of the grid comparing the four groups of study. Same letters within each color indicate no significant differences. (**A**–**F**) represent the different regions in which the defect is divided from R1 to R6.

By region, in R1, defects treated with Zn-HOOC-Si-Membranes attained the highest NBA values, and defects treated with Dox-Si-Membranes and the sham group had the lowest values (Table S3). The OA achieved the highest values in the sham group, and the STA was higher in defects treated with Dox-Si-Membranes and in the sham group (Figure 9A). In R2, defects treated with both HOOC-Si-Membranes and Zn-HOOC-Si-Membranes showed the highest NBA and the lowest STA values. The OAs were similar in all the groups (Figure 9B). In R3, HOOC-Si-Membranes promoted the highest NBA values, and the lowest values were obtained in the sham group. The OA was significantly higher in samples treated with HOOC-Si-Membranes in comparison with the rest of the groups, and the highest values of STA were achieved in samples treated with Dox-Si-Membranes (Figure 9C). In R4, specimens treated with Zn-HOOC-Si-Membranes achieved the highest NBA values, and the lowest values were obtained in the sham group. The OAs were similar in all the groups, and the sham group attained the highest STA values (Figure 9D). In R5, the NBA, OA, and STA performed similarly in all the groups (Figure 9E). In R6, both the NBAs and OAs were similar in all the groups. The lowest STA values were presented in samples treated with Dox-Si-Membranes (Figure 9F).

**Figure 10:**
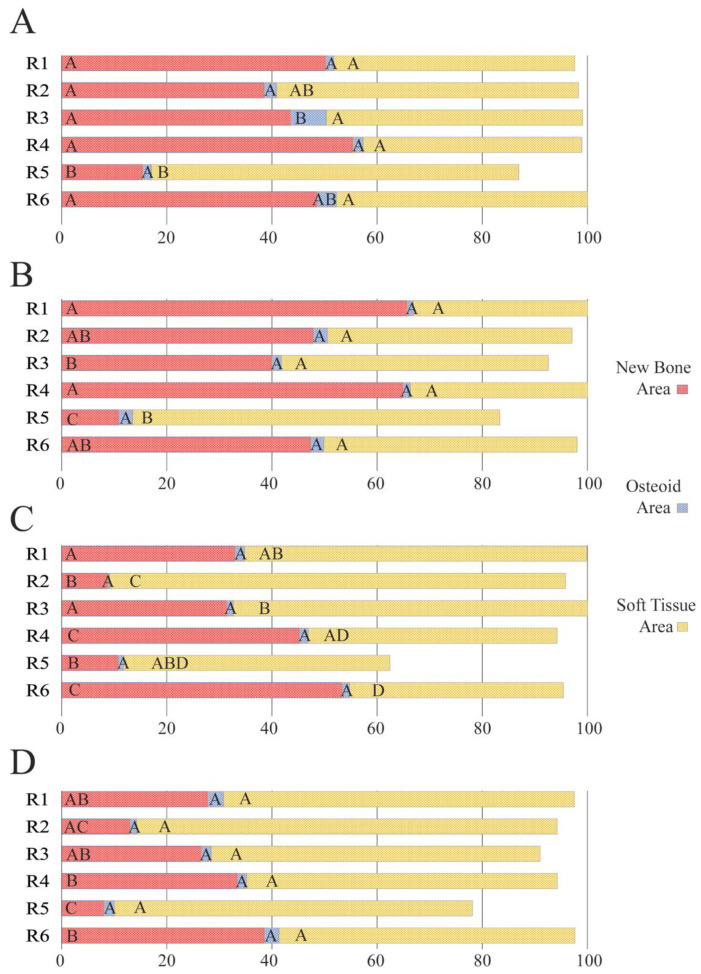
Histomorphometric study of the new bone area (NBA, in red), osteoid area (OA, in blue), and soft tissue area (STA, in yellow) fractions in each of the six regions of the grid. The histomorphometry promoted in each group was assessed when the HCOO-Si-Membranes (**A**), Zn-HCOO-Si-Membranes (**B**), Dox-HCOO-Si-Membranes (**C**) and the sham groups (**D**) were analyzed. Same letters within each color indicate no significant differences.

When the influence of the membranes was analyzed by region, the defects treated with HOOC-Si-Membranes attained the lowest NBA values and the highest STA values in R5 (bottom central); in the other regions, the NBA and STA were similar, in general (Table S4). The highest OA value appeared in R3 (top right) (Figure 10A). R5 showed the lowest NBA values and the highest STA values when Zn-HOOC-Si-Membranes were used. The OAs were similar in all the groups (Figure 10B). The samples treated with Dox-HOOC-Si-Membranes showed the lowest values of NBA in R2 and R5 (central areas) and the highest values in R4 (bottom left) and R6 (bottom right). The OAs were similar in all the groups. The highest STA value was observed in R2 (Figure 10C). The sham group showed the lowest NBA value in R5 without significant differences from R2. The highest values appeared in R4 and R6. The OAs and STAs were similar in all the groups (Figure 10D).

## 4. Discussion

Based on histomorphometric criteria for GBR in calvarial bone defects, a higher proportion of new bone was formed at the periphery compared with the bone found centrally at the defects. The bone regeneration was similar in zones adjacent to both the *dura mater* (bottom) and the polymeric membrane (top), except when doxycycline was used for membrane doping, which showed higher new bone area values in the contiguous zone to the *dura mater*.

New bone formed centrally vs. peripherally should be quantified separately because the mechanism of bone formation for each area appears to be different [1]. Advanced, differentiated, functional bone marrow was observed in all the samples, mainly located at the defect borders (Figure 3A, Figure 4A, Figure 5A, and Figure 7), in agreement with Schallet et al. (2020) [23], except in the sham group (Figure 6A). In the present research, the histology and histomorphometry were performed at six weeks after membrane placement, but at three days, the formation of a few islets of new bone and osteoid was observed at the edges of the defects. At 21 days, a substantial amount of bone was regenerated [2] and ingrowth of the newly formed woven bone was detected at the boundaries of the pristine bone [23]. Pericranial contact in mature animals promotes peripheral osteogenesis, and the majority of bone formation within the defects represented peripheral new bone [1]. This greater bone growing observed at the periphery of the defects corresponded with mature bone marrow that included the presence of well-developed osteons (Figure 4D) and interstitial lamellae, indicating an advanced stage of bone modeling and remodeling [23].

In contrast, the formation of the central bone was mainly attributed to the contact of the defects with the *dura* [1]. Central bone production indicates strong osteogenic influence from the calvarial edge [1] and is likely to be the most challenging region for bone formation compared to the defect limits [2]. Differences between central and peripheral new bone in the osteogenesis of cranial defects were also perceived by Gosain et al. (2003) [1], who reported that reossification of cranial calvarial defects occurred when bone regenerated from the cut edges of the bone and from islands of bone within the *dura mater,* the latter being the most influential. The different patterns of bone distribution could be explained by the release of Si, doxycycline, or Zn, which were assumed to spread out in the defects in concentration gradients with optimal levels at the periphery and detrimental levels at the center [5,6]. Therefore, the null hypothesis must be accepted.

The highest percentage of new bone that was formed in either the central and lateral or peripheral areas of the defects treated with HOOC-Si-Membranes and Zn-HOOC-Si-Membranes in comparison to Dox-HOOC-Si-Membranes (Figure 7) could be due to higher stability and mechanical properties [2,13]. The higher loss modulus that both Zn-HOOC-Si-Membranes and HOOC-Si-Membranes have demonstrated when compared with Dox-HOOC-Si-Membranes [14] could have resulted from a reduced collapse of both the membrane and the overlying soft tissue centrally into the defect [2]. The collapse of a membrane into an osseous defect (Figure 5A) causes less space availability for bone regeneration, limiting the amount of new bone formation [8]. The well-established osteoinductive role of zinc and silica [22] provides one plausible explanation for the enhanced bone regeneration in the central region of the defects treated with both membranes in comparison with those animals treated with Dox-HOOC-Si-Membranes (Figure 7).

Though bone formation and osteoid induction in alveolar bone after doxycycline administration have been reported [24], in the present research, the NBA value was lowest among the animals treated with membranes, although in the peripheral zone of the defects, this group obtained more new bone than the control group (Figure 5A and Figure 7). The use of doxycycline promoted new bone formation mainly in the bottom and peripheral regions (Figure 10C) of the defects, which was representative of an early stage in neoformation [18]. Thus, the bone area fraction increased in the bottom zone of the defect compared with the top zone, and the soft tissue area was reduced from the top to the bottom zones of the defects (Figure 8). The presented membranes were functionalized in an attempt to potentiate not only osteogenic responses [16] but to increase the mineralized and osteoid new bone formation [17]. Osteoid bone is type I collagen when first deposited and not yet mineralized [25]. In the biomineralization process, the production of collagen plays a major role. Type I collagen is expressed in high levels at the end of the proliferative osteoblast state and during the period of matrix deposition [15,26]. Type I collagen is the principal component of the bone extracellular matrix when the osteoblast maturation process occurs. It can be observed as a homogeneous clear blue fringe between the aligned osteoblasts and the mature bone, and its proportion was similar in all the groups (Figure 3B, Figure 4B, Figure 5B, and Figure 6B).

Interstitial connective tissue and areas of trabecular bone formation were observed in all samples (Figure 3A, Figure 4A, Figure 5A, and Figure 6A). Bone regeneration in calvarial defects is unique because of the soft tissue environment of the calvaria. The adjacent pericranium lines the outer cortical table of the skull and *dura* lines the inner [1]. Incomplete occlusion of the surrounding intracranial (dural) and extracranial soft tissues has been associated with impaired bone formation [8] and the blockage of bone remodeling [27]. In animals treated with the Dox-HOOC-Si-Membranes, the impairing of block remodeling was seen in the bone-growing of the inner tabula over the encephalic cavity (Figure 5D). The autocrine and paracrine effects of transforming growth factor β-1 and fibroblast growth factor-2 on the biology of the *dura mater* have been determined [1]. Doxycycline could indirectly prevent the activation of cytokines by inhibiting matrix metalloproteinase (MMP) activities, such as transforming growth factor β1 (TGF-β1) and connective tissue growth factor (CTGF) that might be associated with the BMP-2 signaling of bone formation [28]. In the presence of doxycycline, the reduction of BMP-2 has been demonstrated [29,30]. Moreover, low bone density is associated with poor bone quality, which means a weak bone tissue ensemble of architectural properties and structure, leading to further bone resorption [31]. Newly formed bone tissue may have a lower mineral content than pre-existing, older bone tissue, but regular collagen production was assessed [32] (Figure 5). The sham defects, as well as defects treated with the Dox-HOOC-Si-Membranes, were filled with more fibrous or adipose-like tissue than the rest of the defects in the peripheral zones, although in the central zones of the defects, all the groups performed similarly (Figure 7). Adipocyte-like surrounding tissues and a few immature bone trabeculae, especially in the central areas, were also shown, particularly in the sham group (Figure 6A,B,D). The STA is characteristic of immature tissue [17,33].

Multiple factors influence the bone-healing process, making the GBR of critical defects complex and poorly understood. Among these factors are the osteogenic properties of the adjacent *dura mater*, pericranium, and mesenchymal growth. The periosteum provides both blood supply to cortical bone and osteoprogenitor cells for bone regeneration. The *dura* has been demonstrated to promote reossification of calvarial defects [1]. As already shown in the present study, bone formation was promoted both in the outer layer (under the membrane) and in the inner layer (over *dura mater*). When the top and bottom regions of the defect were compared, histological observations revealed a greater degree of bone formation at the top for those membranes that were considered bioactive because of an osteoinductive effect (i.e., HOOC-Si-Membranes and Zn-HOOC-Si-Membranes). Increased bone formation, especially in the R2 region, demonstrated the active participation of the aforementioned membranes in the regenerative process instead of being a passive barrier [15]. Though the top and bottom NBA values were similar in the sham group, both values were lower than in the membrane groups (Figure 8). The sham group showed the lowest new bone formation among the groups (Figure 7), leading to a lower overall degree of bone restitution in the defect (Figure 6A). This complies with a smaller amount of bone and the relative collapse of the defect in the absence of a membrane. A higher proportion of bone was also obtained by Turri et al. (2016) [18] at the top level of the membrane defect compared with the top level of the sham defect. At the top level of the defect, the membrane compartment might play a role in boosting a natural osteoinductive signal that rapidly promotes bone formation [18]. Comparisons are difficult, as numerous factors may explain the observed differences in calvarial healing studies where dural and pericranial membranes are placed adjacent to calvarial defects. These differences include type of membranes, animal model, age of the animal used [1], and time of healing. Thus, Schaller et al. (2020) [23] observed new bone in the central area of the defect, but after three months of healing in most of the regions, the newly formed bone was next to the *dura mater* rather than on the periosteal sides. The healing period following GBR application is a crucial factor influencing the amount of experimental neogenic bone formation [8]. We speculate that the time point (6 week) may have influenced this result, as Martínez et al., (2014) [3] stated that bone formation at the inner layer of the defect takes place in later periods of healing.

Studies should quantify bone regeneration histometrically instead of subjectively evaluating healed vs. unhealed defects. The present report indicated that the results for optimizing bone regeneration within calvarial defects varied on the basis of the type of membrane barrier used. In the present study, it was found that bone regeneration within critical defects (8 mm in diamenter) in rabbit calvarial over 6 weeks was optimized after the placement of HOOC-Si-Membranes and Zn-HOOC-Si-Membranes. To the best of our knowledge, this was the first study to elucidate the potential of HOOC-Si-Membranes and Zn-HOOC-Si-Membranes to enhance osteogenesis compartmentalized by region in the bone defect for GBR. This research was promising but preliminary, and some limitations need to be addressed before clinical application. For example, the use of different time points further than 6 weeks, an increase in the sample size used (six rabbits), and the use of a positive control should be considered for future action. Related to the period of healing (6 weeks), we preferred to concentrate the full power of the study on the intermediate stages of healing where the effects of modifications in improving healing should have become apparent, compared to normal healing in the body. At a later date, the complete healing of the bone in a standard way may possibly hide, for the most part, the improvement produced by our modifications. Concerning the number of animals, it should be stressed that this limited number of specimens was adequate to identify significant differences, as long as there was a significant change in bone healing. This is in line with the sample sizes used in other publications from other teams. Two more items should be taken into account for the future of the research employing large animal models including the application of experimental membranes for guided tissue regeneration. Aiming to verify bone regeneration engineering efficiency for clinical applications, in vitro studies should required focus on molecular mechanisms and biomarker analyses. In a rabbit model, several limitations that jeopardize the methodology were encountered with the lack of fixation devices to immobilize the clot and potential bone damage due to high animal mobility [34]. Finally, the combined application of zinc and silica in Zn-HOOC-Si-Membranes made it difficult to interpret the outcomes in terms of identifying the component most strongly related to the positive results of the attained bone regeneration or if there existed a combined action of both elements. To reconstruct the maxillofacial skeleton, our results support future studies to develop novel alternatives in guided bone regeneration to maintain aesthetic and function and to contribute to implant survival.

## 5. Conclusions

New bone formation under a guided bone regeneration membrane should also be analyzed separately in specific regions of a defect, as the results encountered in the entire defect may mask possible differences between membranes.

The peripheral regions of the bone defects showed higher osteogenic potential than the central areas when a nanostructured membrane was present, except in the sham group, which showed no differences. Zones adjacent to both the dura mater and the membrane within the calvarial defects showed similar osteogenic potentials, except when HOOC-Si-Membranes and Zn-HOOC-Si-Membranes were present, which promoted higher new bone area values in the center of the defects and under the membranes. HOOC-Si-Membranes promoted the highest proportion of osteoid area in the peripheral-top regions of the defect, and R3 attained the highest proportion among the regions. Among the calvarial defects treated with Dox-HOOC-Si-Membranes, adipose-like tissue concentrated more in the top-central areas, revealing the regions with the least osteogenic potential. Meanwhile, the rest of the groups performed similarly in any location.

## Data Availability

The data presented in this study are available on request from the corresponding author.

## Supplementary Materials

The following supporting information can be downloaded at: https://www.mdpi.com/article/10.3390/polym14102078/s1, Table S1: Data from histomorphometric study of the new bone area, osteoid area, and soft tissue area fractions when lateral and central areas of the defect were compared; Table S2: Data from histomorphometric study of the new bone area, osteoid area, and soft tissue area fractions when top and bottom areas of the defect were compared; Table S3: Data from histomorphometric study of the new bone area, osteoid area, and soft tissue area fractions at each of the six regions of the grid, comparing the four groups of study; Table S4: Data from histomorphometric study of the new bone area, osteoid area, and soft tissue area fractions at each of the six regions of the grid.
