# Supplementary material for: Histomorphometric Analysis of Differential Regional Bone Regeneration Induced by Distinct Doped Membranes
Source: Polymers (Basel). 2022 May 19;14(10):2078. doi: 10.3390/polym14102078 (PMC9147672; doi:10.3390/polym14102078)
Supplement: Supplementary file 1 [file polymers-14-02078-s001.zip › polymers-1706418-supplementary.pdf]

Supplementary Information

Histomorphometric analysis of differential regional bone regeneration induced by distinct doped membranes.

Manuel Toledano [1], Cristina Vallecillo [1], Aida Gutierrez-Corrales [2], Daniel Torres-Lagares [2], Manuel Toledano-Osorio [1*], María-Angeles Serrera-Figallo [2]

[1] Faculty of Dentistry, Colegio Máximo de Cartuja s/n, University of Granada, 18071 Granada, Spain; toledano@ugr.es (M.T.); cvallecillorivas@hotmail.com (C.V.)
[2] Faculty of Dentistry, Oral Surgery Section, University of Sevilla, Avicena s/n, 41009 Sevilla, Spain; agcorrales@us.es (A.G.-C.); danieltl@us.es (D.T.-L.); maserrera@us.es (M.-A.S.-F.)
* Correspondence: mtoledano@ugr.es; Tel.: +34-958-243-789

Table of content:

Table S1: Data from histomorphometric study of the new bone area, osteoid area, and soft tissue area fractions when lateral and central areas of the defect were compared.
Table S2: Data from histomorphometric study of the new bone area, osteoid area, and soft tissue area fractions when top and bottom areas of the defect were compared.
Table S3: Data from histomorphometric study of the new bone area, osteoid area, and soft tissue area fractions at each of the six regions of the grid, comparing the four groups of study
Table S4: Data from histomorphometric study of the new bone area, osteoid area, and soft tissue area fractions at each of the six regions of the grid.

Table S1: Data from histomorphometric study of the new bone area, osteoid area, and soft tissue area fractions when lateral and central areas of the defect were compared.

			Means	SD	SE	LL	UP	Min	Max
NBA	HOOC-Si-Membrane	Central	26.8970	27.91951	4.11651	18.6059	35.1880	.00	100.00
		Pheripheral	49.3903	21.58217	2.25010	44.9208	53.8599	.55	113.13
	Zn-HOOC-Si-Membrane	Central	29.3129	34.21854	4.93902	19.3769	39.2489	.00	99.02
		Pheripheral	54.4649	26.95327	2.75091	49.0037	59.9261	.00	120.15
	Dox-HOOC-Si-Membrane	Central	9.7317	16.04759	2.31627	5.0719	14.3914	.00	54.72
		Pheripheral	40.7239	22.44788	2.29108	36.1755	45.2722	.00	100.08
	Sham	Central	10.4680	21.51848	3.17273	4.0778	16.8582	.00	81.27
		Pheripheral	31.5384	22.41311	2.33673	26.8967	36.1800	.00	90.38
OA	HOOC-Si-Membrane	Central	2.1124	3.09393	.45618	1.1936	3.0312	.00	14.51
		Pheripheral	3.5452	7.07076	.73718	2.0809	5.0095	.00	61.75
	Zn-HOOC-Si-Membrane	Central	2.7106	6.28837	.90765	.8847	4.5366	.00	32.74
		Pheripheral	1.7495	2.37767	.24267	1.2677	2.2312	.00	15.65
	Dox-HOOC-Si-Membrane	Central	.7290	2.17276	.31361	.0981	1.3599	.00	13.14
		Pheripheral	1.5727	1.71730	.17527	1.2248	1.9207	.00	8.05
	Sham	Central	1.6786	3.15008	.47489	.7209	2.6363	.00	12.96
		Pheripheral	2.4049	3.43193	.35780	1.6942	3.1156	.00	27.06
STA	HOOC-Si-Membrane	Central	63.6120	32.56648	4.80166	53.9409	73.2830	.00	100.00
		Pheripheral	45.9774	20.16438	2.10228	41.8015	50.1533	.00	89.11
	Zn-HOOC-Si-Membrane	Central	58.1812	38.88540	5.61262	46.8901	69.4724	.00	100.00
		Pheripheral	42.2261	25.06372	2.55805	37.1478	47.3045	.00	100.00
	Dox-HOOC-Si-Membrane	Central	68.6729	38.93682	5.62005	57.3668	79.9790	.00	100.00
		Pheripheral	55.0939	23.11064	2.35872	50.4112	59.7765	.00	100.00
	Sham	Central	73.7759	37.31295	5.62514	62.4317	85.1201	.00	100.00
		Pheripheral	61.1643	22.46363	2.34200	56.5123	65.8164	.00	100.00

SD: Standard Deviation; SE: Standard Error; LL: Lower Limit; UP: Upper Limit; Min: Minimum; Max: Maximum; NBA: New Bone Area; OA: Osteoid Area; STA: Soft Tissue Area.

Table S2: Data from histomorphometric study of the new bone area, osteoid area, and soft tissue area fractions when top and bottom areas of the defect were compared.

			Means	SD	SE	LL	UP	Min	Max
NBA	HOOC-Si-Membrane	Top	44.0687	26.92393	3.24126	37.6009	50.5365	.00	113.13
		Bottom	39.7164	25.15646	3.02848	33.6731	45.7596	.00	100.00
	Zn-HOOC-Si-Membrane	Top	51.1442	30.65623	3.61287	43.9403	58.3480	.00	117.09
		Bottom	41.0176	32.25652	3.80147	33.4377	48.5975	.00	120.15
	Dox-HOOC-Si-Membrane	Top	24.3776	21.91464	2.58267	19.2280	29.5273	.00	100.08
		Bottom	36.4086	26.90692	3.17101	30.0858	42.7314	.00	89.06
	Sham	Top	22.4130	21.09944	2.54007	17.3444	27.4817	.00	78.75
		Bottom	26.6168	26.92120	3.24093	20.1496	33.0840	.00	90.38
OA	HOOC-Si-Membrane	Top	3.6094	7.85056	.94510	1.7235	5.4953	.00	61.75
		Bottom	2.5258	3.45536	.41598	1.6957	3.3559	.00	15.60
	Zn-HOOC-Si-Membrane	Top	1.9242	3.40952	.40182	1.1230	2.7254	.00	25.62
		Bottom	2.2156	4.74231	.55889	1.1012	3.3299	.00	32.74
	Dox-HOOC-Si-Membrane	Top	1.1622	1.49749	.17648	.8103	1.5141	.00	5.59
		Bottom	1.4207	2.26177	.26655	.8892	1.9522	.00	13.14
	Sham	Top	2.1182	2.49897	.30530	1.5087	2.7278	.00	10.59
		Bottom	2.2201	4.02508	.48456	1.2532	3.1871	.00	27.06
STA	HOOC-Si-Membrane	Top	50.6496	25.71327	3.09551	44.4726	56.8266	.00	100.00
		Bottom	53.0616	26.87195	3.23500	46.6062	59.5169	.00	100.00
	Zn-HOOC-Si-Membrane	Top	44.1233	29.63989	3.49309	37.1583	51.0884	.00	100.00
		Bottom	50.9657	32.46473	3.82600	43.3369	58.5945	.00	100.00
	Dox-HOOC-Si-Membrane	Top	73.0385	23.59752	2.78099	67.4933	78.5836	.00	100.00
		Bottom	46.2019	29.65358	3.49471	39.2337	53.1702	.00	100.00
	Sham	Top	69.4299	25.01630	3.05623	63.3279	75.5318	.00	100.00
		Bottom	61.1806	31.36690	3.77613	53.6454	68.7157	.00	100.00

SD: Standard Deviation; SE: Standard Error; LL: Lower Limit; UP: Upper Limit; Min: Minimum; Max: Maximum; NBA: New Bone Area; OA: Osteoid Area; STA: Soft Tissue Area.

Table S3: Data from histomorphometric study of the new bone area, osteoid area, and soft tissue area fractions at each of the six regions of the grid, comparing the four groups of study

		Means	SD	SE	LL	UP	Min	Max
R1								
NBA	HOOC-Si-Membrane	50.2135	27.98468	5.83521	38.1120	62.3150	8.74	113.13
	Zn-HOOC-Si-Membrane	65.6671	27.92642	5.70046	53.8748	77.4594	.00	117.09
	Dox-HOOC-Si-Membrane	32.9833	23.66917	4.83145	22.9887	42.9779	1.39	100.08
	Sham	27.7887	20.46795	4.26786	18.9377	36.6397	.00	78.75
OA	HOOC-Si-Membrane	1.5704	1.87759	.39150	.7585	2.3824	.00	7.14
	Zn-HOOC-Si-Membrane	1.1988	1.31330	.26808	.6442	1.7533	.00	4.46
	Dox-HOOC-Si-Membrane	1.8454	1.59295	.32516	1.1728	2.5181	.00	5.59
	Sham	3.0274	2.99688	.62489	1.7314	4.3233	.00	10.59
STA	HOOC-Si-Membrane	45.7939	24.90019	5.19205	35.0263	56.5616	.00	89.11
	Zn-HOOC-Si-Membrane	35.0492	25.59266	5.22408	24.2423	45.8560	.00	100.00
	Dox-HOOC-Si-Membrane	65.0717	23.26270	4.74848	55.2487	74.8946	.00	98.61
	Sham	66.6713	21.24426	4.42973	57.4846	75.8580	20.60	100.00
R2								
NBA	HOOC-Si-Membrane	38.4700	30.24636	6.30680	25.3905	51.5495	.00	100.00
	Zn-HOOC-Si-Membrane	47.7871	34.92616	7.12927	33.0391	62.5351	.00	99.02
	Dox-HOOC-Si-Membrane	8.7225	15.51915	3.16783	2.1693	15.2757	.00	54.72
	Sham	12.9296	21.05526	4.39032	3.8246	22.0345	.00	71.42
OA	HOOC-Si-Membrane	2.4352	2.88983	.60257	1.1856	3.6849	.00	11.65
	Zn-HOOC-Si-Membrane	2.7513	5.44616	1.11169	.4515	5.0510	.00	25.62
	Dox-HOOC-Si-Membrane	.3883	1.06816	.21804	-.0627	.8394	.00	4.65
	Sham	1.3010	2.37498	.51826	.2199	2.3820	.00	8.62
STA	HOOC-Si-Membrane	57.3948	29.56988	6.16575	44.6078	70.1818	.00	100.00
	Zn-HOOC-Si-Membrane	46.5375	34.33499	7.00860	32.0391	61.0359	.11	100.00
	Dox-HOOC-Si-Membrane	86.7229	24.15964	4.93157	76.5212	96.9246	.00	100.00
	Sham	80.0143	29.29796	6.39334	66.6780	93.3506	.00	100.00
R3								
NBA	HOOC-Si-Membrane	43.5226	21.68071	4.52074	34.1472	52.8981	.55	97.57
	Zn-HOOC-Si-Membrane	39.9783	23.22653	4.74110	30.1706	49.7860	13.57	100.00
	Dox-HOOC-Si-Membrane	31.4271	17.22120	3.51526	24.1552	38.6990	.00	70.62
	Sham	26.5209	19.28451	4.02110	18.1816	34.8601	.00	73.50
OA	HOOC-Si-Membrane	6.8226	12.72943	2.65427	1.3180	12.3272	.72	61.75
	Zn-HOOC-Si-Membrane	1.8225	1.79615	.36664	1.0641	2.5809	.00	7.21
	Dox-HOOC-Si-Membrane	1.2529	1.45719	.29745	.6376	1.8682	.00	4.37
	Sham	1.9552	1.75953	.36689	1.1943	2.7161	.00	6.67
STA	HOOC-Si-Membrane	48.7600	21.75498	4.53623	39.3524	58.1676	.00	85.41
	Zn-HOOC-Si-Membrane	50.7833	27.19926	5.55203	39.2981	62.2686	.00	86.43
	Dox-HOOC-Si-Membrane	67.3208	17.27310	3.52586	60.0270	74.6146	26.24	100.00
	Sham	62.5243	21.93273	4.57329	53.0399	72.0088	.00	93.35
R4								

NBA	HOOC-Si-Membrane	55.3535	18.72655	3.90476	47.2555	63.4514	25.61	100.00
	Zn-HOOC-Si-Membrane	64.9108	25.54806	5.21498	54.1228	75.6988	21.12	120.15
	Dox-HOOC-Si-Membrane	45.1658	23.20595	4.73689	35.3668	54.9648	.00	81.26
	Sham	33.3322	22.73136	4.73982	23.5024	43.1619	2.85	89.54
OA	HOOC-Si-Membrane	1.9800	2.30973	.48161	.9812	2.9788	.00	9.49
	Zn-HOOC-Si-Membrane	1.4079	2.08255	.42510	.5285	2.2873	.00	8.83
	Dox-HOOC-Si-Membrane	1.7571	1.86676	.38105	.9688	2.5453	.00	5.53
	Sham	1.8113	2.12764	.44364	.8912	2.7314	.00	6.90
STA	HOOC-Si-Membrane	41.6352	17.77971	3.70733	33.9467	49.3237	.00	73.50
	Zn-HOOC-Si-Membrane	34.9158	23.63496	4.82447	24.9357	44.8960	1.80	78.88
	Dox-HOOC-Si-Membrane	47.3167	22.00996	4.49277	38.0227	56.6107	.00	100.00
	Sham	59.1565	22.98074	4.79182	49.2189	69.0941	9.04	94.02
R5								
NBA	HOOC-Si-Membrane	15.3239	19.98853	4.16790	6.6802	23.9676	.00	60.60
	Zn-HOOC-Si-Membrane	10.8388	21.46208	4.38093	1.7761	19.9014	.00	78.27
	Dox-HOOC-Si-Membrane	10.7408	16.83086	3.43559	3.6338	17.8479	.00	54.16
	Sham	8.0065	22.16179	4.62105	-1.5770	17.5900	.00	81.27
OA	HOOC-Si-Membrane	1.7896	3.31827	.69191	.3546	3.2245	.00	14.51
	Zn-HOOC-Si-Membrane	2.6700	7.15139	1.45977	-.3498	5.6898	.00	32.74
	Dox-HOOC-Si-Membrane	1.0696	2.87470	.58680	-.1443	2.2835	.00	13.14
	Sham	2.0235	3.74256	.78038	.4051	3.6419	.00	12.96
STA	HOOC-Si-Membrane	69.8291	34.84481	7.26564	54.7611	84.8972	.00	100.00
	Zn-HOOC-Si-Membrane	69.8250	40.34918	8.23624	52.7870	86.8630	.00	100.00
	Dox-HOOC-Si-Membrane	50.6229	42.83042	8.74272	32.5372	68.7086	.00	100.00
	Sham	68.0800	43.24147	9.01647	49.3810	86.7790	.00	100.00
R6								
NBA	HOOC-Si-Membrane	48.4717	15.74919	3.28393	41.6613	55.2822	21.36	75.47
	Zn-HOOC-Si-Membrane	47.3033	22.61202	4.61566	37.7551	56.8516	.97	85.41
	Dox-HOOC-Si-Membrane	53.3192	18.63343	3.80353	45.4510	61.1874	21.25	89.06
	Sham	38.5117	25.98322	5.41788	27.2758	49.7477	1.91	90.38
OA	HOOC-Si-Membrane	3.8078	4.23813	.88371	1.9751	5.6405	.00	15.60
	Zn-HOOC-Si-Membrane	2.5688	3.59423	.73367	1.0510	4.0865	.00	15.65
	Dox-HOOC-Si-Membrane	1.4354	1.94832	.39770	.6127	2.2581	.00	8.05
	Sham	2.8257	5.56278	1.15992	.4201	5.2312	.00	27.06
STA	HOOC-Si-Membrane	47.7204	15.53770	3.23983	41.0014	54.4394	17.02	77.99
	Zn-HOOC-Si-Membrane	48.1563	20.46178	4.17674	39.5160	56.7965	14.03	98.42
	Dox-HOOC-Si-Membrane	40.6663	18.49097	3.77445	32.8582	48.4743	8.74	77.44
	Sham	56.3052	23.75404	4.95306	46.0332	66.5772	9.62	95.91

SD: Standard Deviation; SE: Standard Error; LL: Lower Limit; UP: Upper Limit; Min: Minimum; Max: Maximum; NBA: New Bone Area; OA: Osteoid Area; STA: Soft Tissue Area.

Table S4: Data from histomorphometric study of the new bone area, osteoid area, and soft tissue area fractions at each of the six regions of the grid.

		Means	SD	SE	LL	UP	Min	Max
HOOC-Si-Membrane								
NBA	R1	50.2135	27.98468	5.83521	38.1120	62.3150	8.74	113.13
	R2	38.4700	30.24636	6.30680	25.3905	51.5495	.00	100.00
	R3	43.5226	21.68071	4.52074	34.1472	52.8981	.55	97.57
	R4	55.3535	18.72655	3.90476	47.2555	63.4514	25.61	100.00
	R5	15.3239	19.98853	4.16790	6.6802	23.9676	.00	60.60
	R6	48.4717	15.74919	3.28393	41.6613	55.2822	21.36	75.47
OA	R1	1.5704	1.87759	.39150	.7585	2.3824	.00	7.14
	R2	2.4352	2.88983	.60257	1.1856	3.6849	.00	11.65
	R3	6.8226	12.72943	2.65427	1.3180	12.3272	.72	61.75
	R4	1.9800	2.30973	.48161	.9812	2.9788	.00	9.49
	R5	1.7896	3.31827	.69191	.3546	3.2245	.00	14.51
	R6	3.8078	4.23813	.88371	1.9751	5.6405	.00	15.60
STA	R1	45.7939	24.90019	5.19205	35.0263	56.5616	.00	89.11
	R2	57.3948	29.56988	6.16575	44.6078	70.1818	.00	100.00
	R3	48.7600	21.75498	4.53623	39.3524	58.1676	.00	85.41
	R4	41.6352	17.77971	3.70733	33.9467	49.3237	.00	73.50
	R5	69.8291	34.84481	7.26564	54.7611	84.8972	.00	100.00
	R6	47.7204	15.53770	3.23983	41.0014	54.4394	17.02	77.99
Zn-HOOC-Si-Membrane								
NBA	R1	65.6671	27.92642	5.70046	53.8748	77.4594	.00	117.09
	R2	47.7871	34.92616	7.12927	33.0391	62.5351	.00	99.02
	R3	39.9783	23.22653	4.74110	30.1706	49.7860	13.57	100.00
	R4	64.9108	25.54806	5.21498	54.1228	75.6988	21.12	120.15
	R5	10.8388	21.46208	4.38093	1.7761	19.9014	.00	78.27
	R6	47.3033	22.61202	4.61566	37.7551	56.8516	.97	85.41
OA	R1	1.1988	1.31330	.26808	.6442	1.7533	.00	4.46
	R2	2.7513	5.44616	1.11169	.4515	5.0510	.00	25.62
	R3	1.8225	1.79615	.36664	1.0641	2.5809	.00	7.21
	R4	1.4079	2.08255	.42510	.5285	2.2873	.00	8.83
	R5	2.6700	7.15139	1.45977	-.3498	5.6898	.00	32.74
	R6	2.5688	3.59423	.73367	1.0510	4.0865	.00	15.65
STA	R1	35.0492	25.59266	5.22408	24.2423	45.8560	.00	100.00
	R2	46.5375	34.33499	7.00860	32.0391	61.0359	.11	100.00
	R3	50.7833	27.19926	5.55203	39.2981	62.2686	.00	86.43
	R4	34.9158	23.63496	4.82447	24.9357	44.8960	1.80	78.88
	R5	69.8250	40.34918	8.23624	52.7870	86.8630	.00	100.00
	R6	48.1563	20.46178	4.17674	39.5160	56.7965	14.03	98.42
Dox-HOOC-Si-Membrane								
NBA	R1	32.9833	23.66917	4.83145	22.9887	42.9779	1.39	100.08
	R2	8.7225	15.51915	3.16783	2.1693	15.2757	.00	54.72
	R3	31.4271	17.22120	3.51526	24.1552	38.6990	.00	70.62
	R4	45.1658	23.20595	4.73689	35.3668	54.9648	.00	81.26
	R5	10.7408	16.83086	3.43559	3.6338	17.8479	.00	54.16
	R6	53.3192	18.63343	3.80353	45.4510	61.1874	21.25	89.06

OA	R1	1.8454	1.59295	.32516	1.1728	2.5181	.00	5.59
	R2	.3883	1.06816	.21804	-.0627	.8394	.00	4.65
	R3	1.2529	1.45719	.29745	.6376	1.8682	.00	4.37
	R4	1.7571	1.86676	.38105	.9688	2.5453	.00	5.53
	R5	1.0696	2.87470	.58680	-.1443	2.2835	.00	13.14
	R6	1.4354	1.94832	.39770	.6127	2.2581	.00	8.05
STA	R1	65.0717	23.26270	4.74848	55.2487	74.8946	.00	98.61
	R2	86.7229	24.15964	4.93157	76.5212	96.9246	.00	100.00
	R3	67.3208	17.27310	3.52586	60.0270	74.6146	26.24	100.00
	R4	47.3167	22.00996	4.49277	38.0227	56.6107	.00	100.00
	R5	50.6229	42.83042	8.74272	32.5372	68.7086	.00	100.00
	R6	40.6663	18.49097	3.77445	32.8582	48.4743	8.74	77.44
Sham								
NBA	R1	27.7887	20.46795	4.26786	18.9377	36.6397	.00	78.75
	R2	12.9296	21.05526	4.39032	3.8246	22.0345	.00	71.42
	R3	26.5209	19.28451	4.02110	18.1816	34.8601	.00	73.50
	R4	33.3322	22.73136	4.73982	23.5024	43.1619	2.85	89.54
	R5	8.0065	22.16179	4.62105	-1.5770	17.5900	.00	81.27
	R6	38.5117	25.98322	5.41788	27.2758	49.7477	1.91	90.38
OA	R1	3.0274	2.99688	.62489	1.7314	4.3233	.00	10.59
	R2	1.3010	2.37498	.51826	.2199	2.3820	.00	8.62
	R3	1.9552	1.75953	.36689	1.1943	2.7161	.00	6.67
	R4	1.8113	2.12764	.44364	.8912	2.7314	.00	6.90
	R5	2.0235	3.74256	.78038	.4051	3.6419	.00	12.96
	R6	2.8257	5.56278	1.15992	.4201	5.2312	.00	27.06
STA	R1	66.6713	21.24426	4.42973	57.4846	75.8580	20.60	100.00
	R2	80.0143	29.29796	6.39334	66.6780	93.3506	.00	100.00
	R3	62.5243	21.93273	4.57329	53.0399	72.0088	.00	93.35
	R4	59.1565	22.98074	4.79182	49.2189	69.0941	9.04	94.02
	R5	68.0800	43.24147	9.01647	49.3810	86.7790	.00	100.00
	R6	56.3052	23.75404	4.95306	46.0332	66.5772	9.62	95.91

SD: Standard Deviation; SE: Standard Error; LL: Lower Limit; UP: Upper Limit; Min: Minimum; Max: Maximum; NBA: New Bone Area; OA: Osteoid Area; STA: Soft Tissue Area.
